# RET kinase alterations in targeted cancer therapy

**DOI:** 10.20517/cdr.2020.15

**Published:** 2020-05-11

**Authors:** Xuan Liu, Xueqing Hu, Tao Shen, Qi Li, Blaine H. M. Mooers, Jie Wu

**Affiliations:** ^1^Peggy and Charles Stephenson Cancer Center, University of Oklahoma Health Sciences Center, Oklahoma City, OK 73104, USA.; ^2^Department of Pathology, University of Oklahoma Health Sciences Center, Oklahoma City, OK 73104, USA.; ^3^Department of Medical Oncology and Cancer Institute, ShuGuang Hospital, Shanghai University of Traditional Chinese Medicine, Shanghai 201203, China.; ^4^Department of Biochemistry and Molecular Biology, University of Oklahoma Health Sciences Center, Oklahoma City, OK 73104, USA.; ^#^Authors contributed equally.

**Keywords:** Rearranged during transfection, protein tyrosine kinase inhibitor, mutation, gene fusion, targeted therapy, acquired resistance

## Abstract

The rearranged during transfection (RET) gene encodes a protein tyrosine kinase. RET alterations by point mutations and gene fusions were found in diverse cancers. RET fusions allow abnormal expression and activation of the oncogenic kinase, whereas only a few of RET point mutations found in human cancers are known oncogenic drivers. Earlier studies of RET-targeted therapy utilized multi-targeted protein tyrosine kinase inhibitors (TKIs) with RET inhibitor activity. These multi-targeted TKIs often led to high-grade adverse events and were subject to resistance caused by the gatekeeper mutations. Recently, two potent and selective RET TKIs, pralsetinib (BLU-667) and selpercatinib (LOXO-292), were developed. High response rates to these selective RET inhibitors across multiple forms of RET alterations in different types of cancers were observed in clinical trials, demonstrating the RET dependence in human cancers harboring these RET lesions. Pralsetinib and selpercatinib were effective in inhibiting RET^V804L/M^ gatekeeper mutants. However, adaptive mutations that cause resistance to pralsetinib or selpercatinib at the solvent front RET^G810^ residue have been found, pointing to the need for the development of the next-generation of RET TKIs.

## Introduction

Rearranged during transfection (RET) is a transmembrane receptor protein tyrosine kinase (PTK) activated normally by forming ternary complexes with its cognate ligands and co-receptors^[1,2]^. The four RET co-receptors, glial cell-derived neurotrophic factor (GDNF) receptor-α family proteins (GFRα1-4), dictate ligand specificity. GDNF, neurturin, artemin, persephin, differentiation factor 15 (GDF15), and GDNF receptor-α (GFRα)-like protein (GFRAL) are the currently known RET ligands^[1-3]^. Mutations and gene fusions could lead to aberrant dimerization, expression, and activation of the RET protein tyrosine kinase^[1,4,5]^. RET point mutations were associated with medullary thyroid cancer (MTC)^[6]^, whereas RET fusions were found in 20% of papillary thyroid carcinoma (PTC)^[7]^ and 1%-2% of lung adenocarcinoma^[8-11]^. However, studies in the last few years have unraveled RET alterations in diverse cancer types^[12-14]^. While RET fusions are oncogenic, the functional significance of many RET point mutations remains to be characterized. RET alterations that result in activated RET kinase are targetable oncogenic events. Earlier efforts utilized multi-targeted TKIs with RET inhibitor activity^[4,15-17]^. These inhibitors are subject to resistance from mutations at the gatekeeper residue of the RET kinase domain. Recently, two highly potent and selective RET TKIs, pralsetinib^[18]^ and selpercatinib^[19]^, were developed. Preliminary results from clinical trials showed remarkable responses to these selective RET TKIs^[20,21]^. The high response rates of pralsetinib and selpercatinib unequivocally established RET as the therapeutic target in human cancers that harbor oncogenic RET-alterations. However, although the selective RET inhibitors are gatekeeper mutant-effective, acquired mutations at the solvent front residue have been found^[22]^. In this article, we critically review recent findings of RET alterations in human cancers, including both oncogenic and TKI-adapted alterations, targeted therapy of RET-altered cancers, and raise issues that require further studies.

## RET alteration in cancers

**Figure 1:**
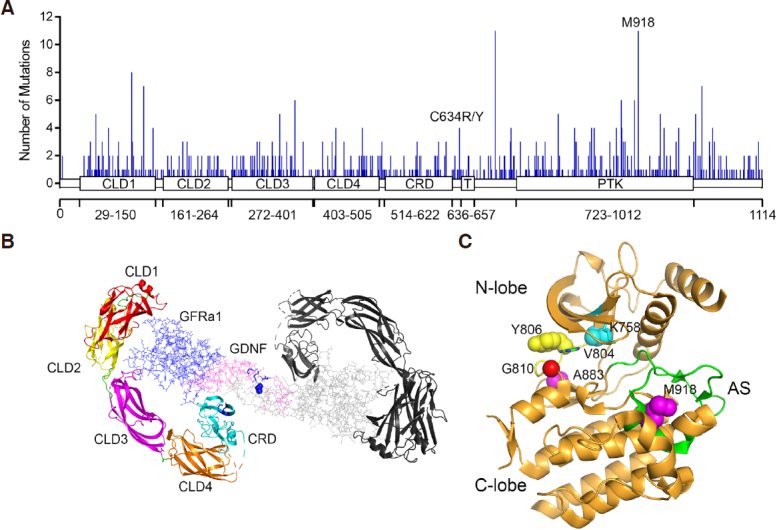
RET missense mutations and structure. A: distribution of missense mutations mapped to the RET protein. Data were based on the curated dataset of cBioportal that contains results from 176 non-redundant studies that have 46,612 samples from 44,284 patients. Locations of the CLD and CRD domains are based on the 3D structure determined by cryo-EM (PDB: 6Q2N)^[3]^; B: structure of RET/GFRα1/GDNF complex based on PDB 6Q2N. Disulfide bonds in CRD and in GDNF are shown in blue; C: the RET kinase domain structure (PDB: 6NJA)^[27]^. AS: activating segment; V804 and K758: gate residues; Y806: hinge residue; G810: solvent front residue; M918 and A883: two of MTC-associated mutation residues; RET: rearranged during transfection; CLD: cadherin-like domains; CRD: cysteine-rich domain; GDNF: glial cell-derived neurotrophic factor

The RET proto-oncogene resides at human chromosome 10q11.21 and has 20 exons. The first 11 exons encode the extracellular and the transmembrane (exon 11) domains. Alternative splicing results in three proteins, RET51, RET9 and RET43, that differ in the lengths of carboxyl-terminal tails^[23]^. RET51 has 1114 amino acid residues [Figure 1A]. The longer RET51 has a stronger transformation activity than that of the shorter RET9^[11]^. The RET extracellular domain has four atypical cadherin-like domains (CLD1-4) and a cysteine-rich domain (CRD) [Figure 1B]. The CRD has seven disulfide bonds. C630 and C634, located in the linker region between the CRD and the transmembrane domain, form another disulfide bone^[3]^. The extracellular domain forms a C-shaped clamp to bind a co-receptor and a ligand^[3]^. CLD1, CLD2, and CLD3 form the interacting face with the co-receptor, while CRD contacts both the co-receptor and the ligand. The ligand-induced dimerization of the receptor/co-receptor/ligand complex activates the receptor^[3,24]^.

Oncogenic gain-of-function RET mutations were originally found in multiple endocrine neoplasia type 2 (MEN2). MEN2 has three clinical subtypes: MEN2A, MEN2B and familial medullary thyroid carcinoma (FMTC)^[1,23]^. MEN2A-associated RET mutations occur most often in the extracellular regions, particularly disulfide bond-forming C609, C611, C618, C620, C630, and C634 residues located in the CRD domain and the region connecting the CRD and the transmembrane domains^[1,4,23]^. It is believed that mutations in these Cys residues disrupt the intramolecular disulfide bonds, allowing formation of abnormal intermolecular disulfide bonds, which lead to receptor dimerization and kinase activation. In the RET/co-receptor/ligand complexes (for instance, the RET/GFRα1/GDNF complex shown in Figure 1)^[3]^, the distance between two receptor molecules in any dimer of these complexes is too far for intermolecular disulfide bond formation between the Cys residues located within the CRD. Thus, based on the RET/co-receptor/ligand complex structures (PDB: 6Q2N, 6Q2S, 6Q2J, 6Q2R, 6Q2O), RET ligands appear to act as inhibitors for abnormal formation of intermolecular disulfide bonds between the C609, C611, C618 or C620 residues within the CRD. Thus, the ligand-independent dimerization via formation of intermolecular disulfide bond between Cys residues in the CRD domain may actually require the absence of the ligand. It is possible that C630 and C634 located in the linker region between the CRD and the transmembrane domains are in a closer proximity between two RET protein molecules in the receptor/co-receptor/ligand complexes, allowing formation of an intermolecular disulfide bond. However, the extracellular domain of RET or RETC634R could not form dimers in solution^[25]^. It was reported that a fraction of RET^C634R/Y^ expressed in NIH3T3 cells formed dimers under non-reducing conditions^[26]^. Whether the C634R/Y-mediated dimerization is facilitated by a co-receptor, the transmembrane domain, or the intracellular domain remains unknown. Hereditary and somatic RET mutations in the more aggressive MEN2B, such as M918T, A883F, V804M/Y806C, V804M/E805K, V908M/S904C, are located in the cytoplasmic kinase domain. M918T and S904 are located in the activating segment [Figure 1C]. M918T is the strongest transforming mutant *in vitro* and is associated with the most aggressive tumors^[1]^. V804 is the gatekeeper residue [Figure 1C]. Y806 is a hinge residue in the ATP binding pocket. Mutations in these kinase domain residues may increase ATP binding affinity and/or alter autophosphorylation^[5]^. Mutations in both extracellular and intracellular RET domains are found in FMTC. FMTC-associated mutations may have milder effects on the RET kinase^[5,23]^.

Figure 1A shows the distribution of 701 missense RET mutations from the curated set of 176 non-redundant studies in cBioportal^[12,28]^. These mutations occurred in 413 positions located throughout the molecule. Although these mutations were found in various types of cancers, only a few of these mutations have been annotated functionally. One may speculate that most of these variants of unknown significance (VUS) were passenger mutations that did not have a functional role in the tumors. For instance, if a mutated gene was not expressed, the mutation could be considered a silent variant without a function. However, examination of the available expression data of these RET mutants in the tumors in which they were identified showed that most of the mutated RET genes were expressed.

Gene rearrangements that led to fusion RET proteins, on the other hand, were found in 20%-40% of PTC and 1%-2% of lung adenocarcinoma^[1,4]^. Unlike missense substituting mutations that could occur either hereditarily or somatically, and noting that the functional consequences of many mutations are unknown, RET gene fusion occur only somatically and the fusion partners contain protein dimerization domains^[4]^. Consequently, the fusions RET proteins are aberrantly activated via dimerization induced by the fusion partners. Moreover, the fusion partners direct the expression of the fusion oncogenes in those cell types in which RET and/or its co-receptors are not normally expressed^[4]^. This allows oncogenic activation of RET fusions in cell types that the full-length RET mutants cannot accomplish. The fusion partners also alter the cellular location of the RET kinase, allowing fusion kinases to form different signaling hubs^[29,30]^. With exceptions, RET fusions often occur via breakpoints within intron 11, resulting in fusion proteins containing RET intracellular fragments encoded by exons 12-20 (E713 to the C-terminal residue) [Figure 1]. Table 1 lists 74 RET fusions found in 10 tissue types in the curated set of non-redundant studies of cBioportal. Consistent with other reports^[4,9,11,31-33]^, the intrachromosomal rearrangements^[34]^ CCDC6-RET and NCOA4-RET occurred most often in PTC, whereas the intrachromosomal rearrangements KIF5B-RET and CCDC6-RET were the most common in lung adenocarcinoma.

**Table 1:** RET fusions in the TCGA curated set of 176 non-redundant studies

RET fusion	Tumor type*										Total
	PTC	LuAd	Thy	Colon	Breast	LuSc	Pro	Ova	Stom	AML	
CCDC6-RET	24	4	1	1				1	1		32
KIF5B-RET		17				1					18
NCOA4-RET	5	1		1			1				8
ERC1-RET	2				1						3
FKBP15-RET	1									1	2
AKAP13-RET	1										1
DLG5-RET	1										1
PDCD10-RET			1								1
RASSF4-RET		1									1
CUBN-RET		1									1
MRLN-RET	1										1
TBL1XR1-RET	1										1
TFG-RET			1								1
TRIM27-RET	1										1
TRIM33-RET		1									1
SPECC1L-RET	1										1
Subtotal	38	25	3	2	1	1	1	1	1	1	74

*LuAd: lung adenocarcinoma; Thy: poorly differentiated thyroid; LuSc: lung squamous; Pro: prostate; Ova: ovarian; Stom: stomach; AML: acute myeloid leukemia; RET: rearranged during transfection; TCGA: The Cancer Genome Atlas

Using hybrid capture targeted genomic profiling of RET introns 9-11 and all exons, a study in 9693 cases of breast cancer identified 121 RET alterations^[35]^. Among these were eight activating RET fusions (CCDC6-RET, NCOA4-RET, RASGEF1A-RET) and another eight cases of RET gene rearrangements (tandem duplication, intergenic space, and two other cases of potential partner genes). A metastatic, estrogen receptor- and HER2-targeted therapy refractory breast cancer was found to harbor the NCOA4-RET fusion. Combination of the multi-targeted RET TKI cabozantinib in the subsequent treatment regimen resulted in tumor reduction^[35,36]^.

In addition to analyzing tumor tissue samples, Rich *et al.*^[14]^ analyzed NGS data of circulating cell-free tumor DNA (cfDNA) from 32,989 patients with metastatic solid tumors (Guardant Health). One hundred seventy-six known and predicted oncogenic alterations were found in 170 patients. These include 143 in-frame fusions and 33 missense mutations. In addition, VUS were identified in 529 patients. RET fusions were found in 116 cases of NSCLC, 14 cases of colorectal cancer, 2 cases of breast cancer, 1 case of thyroid cancer, and 3 cases of unknown primary cancer. Among other mutations, M918T and CRD mutations were found in six cases of thyroid cancer and five cases of breast cancer.

## RET protein tyrosine kinase inhibitors

### Targeting RET fusion^+^ NSCLC with multi-targeted TKIs with RET inhibitor activity

Soon after the discovery of RET fusions in NSCLC^[8-11]^, Drilon *et al.*^[15,37]^ conducted the first prospective phase 2 clinical trial of treating RET fusion^+^ NSCLC patients with cabozantinib. The overall response rate (ORR) to cabozantinib in RET fusion^+^ NSCLC was 28% (7 of 25 patients). This ORR was higher than the ORRs of cabozantinib in unselected NSCLC patients (10%), the chemotherapy drugs pemetrexed (9%) and docetaxel (8%), or the immune checkpoint blockage agent nivolumab (20%). No co-mutation of known cabozantinib targets was found in these cabozantinib-responsive tumors. However, the ORR was lower than that of the ALK inhibitors (57%) or ROS1 inhibitors (72%) in NSCLC patients with ALK or ROS1 fusions. No patient had a complete response to cabozantinib^[15]^. However, in a retrospective analysis of a global, multicenter network registry of 19 RET fusion^+^ NSCLC patients, one complete response to cabozantinib (5%) was recorded^[38]^. Most of the patients (73%) required dose reduction due to drug-related toxicities. The median progression-free survival (PFS) was 5.5 months. However, durable response was observed in one patient who had been treated with cabozantinib for more than 3 years at the time of reporting.

Two prospective phase 2 trials of vandetanib in RET fusion^+^ NSCLC were conducted in East Asia^[16,17]^. The ORR was 18% in the Korean trial^[16]^, whereas it was 53% in the Japanese trial^[17]^. The median PFS was 4.5-4.7 months in these two studies. In the global, multicenter registry analysis, the ORR to vandetanib was 18%, and the median PFS was 2.9 months. Four patients (23%) required dose reduction in the Korean trial, while 10 patients (53%) patients required dose reduction in the Japanese trial.

The results of a multicenter, phase 2 trial of lenvatinib in RET fusion^+^ NSCLC were reported recently^[39]^. The ORR was 16% (4/25), and the median PFS was 7.3 months. Twenty-three (92%) patients had a drug-related adverse event and six (24%) patients discontinued the lenvatinib treatment due to drug-related toxicities. Responses to other multi-targeted TKIs sunitinib, alectinib, and nintedanib in RET fusion^+^ NSCLC were documented in analyses of retrospective clinical data^[38,40]^. Interestingly, in addition to the above mentioned complete response of a patient to cabozantinib treatment, one patient who received nintedanib had complete response^[38]^. Furthermore, stable disease was observed in patients treated with sorafenib and ponatinib^[38,40]^.

It is believed that the adverse events induced by the multi-targeted RET TKIs were in part due to VEGFR inhibition^[4,15]^. For instance, cabozantinib inhibits RET with an IC50 of 5.2 nM, but it inhibits VEGFR2 with an IC50 of 0.04 nM. Agerafenib (RXDX-105) was identified as a multi-targeted RET TKI (IC50: 0.33 nM) that spared VEGFR1 (IC50: 140 nM) and VEGFR2 (IC50: 257 nM)^[41,42]^. Agerafenib was originally identified as a BRAF inhibitor but kinase profiling revealed its RET inhibitor activity. In a phase I/Ib clinical trial of agerafenib, the recommended phase 2 dose was selected at 275 mg once a day. At this dose, agerafenib had a steady state plasma level above the target thresholds of inhibiting RET and BRAF, but below that of inhibiting VGEFR2^[43]^. In the phase Ib cohort, no response was observed in prior RET inhibitor (cabozantinib, vandetanib)-treated patients. In RET inhibitor-naïve NSCLC patients, the ORR was 19% (6/31) and stable disease was achieved in 39% (12/31) of patients. Notably, none of the responders had KIF5B-RET fusion although stable disease was achieved in agerafenib-treated patients with KIF5B-RET fusion^+^ tumors. In non-KIF5B RET fusion tumors, the ORR was 67%. The mechanism underlying the difference in response to agerafenib between KIF5B-RET fusion and non-KIF5B RET fusion remains elusive.

### RET-selective kinase inhibitors

Pralsetinib and selpercatinib are the new generation of RET-selective TKIs that were recently developed and rapidly progressed to clinical trials^[18,19]^. Pralsetinib has 87-fold selectivity against VEGFR2 and 20-fold selectivity against JAK1^[18,20]^. Selpercatinib has > 100-fold selectivity against VEGFR2^[44]^. In addition to being highly selective for RET, both pralsetinib and selpercatinib are effective in inhibiting the RET^V804L/M^ gatekeeper mutants^[18-20,44]^. Another important property of these drugs is that they are effective in the central nervous system. Intracranial metastases occur frequently in RET fusion^+^ NSCLC^[40]^.

In the phase 1/2 trial of pralsetinib (NCT03037385) in patients with thyroid, NSCLC, and other advanced solid tumors (ARROW), treatment-related adverse events were mostly grade 1/2 at the chosen phase 2 dose of 400 mg QD. The ORR was 58% and the disease control rate was 96% in RET fusion^+^ NSCLC patients (*n* = 48) regardless of prior treatments (chemotherapy, PD-1 or PD-L1 inhibitor, multi-targeted TKIs with RET inhibitor activity)^[20]^. In MTC (*n* = 32), the ORR was 56% and the disease control rate was 94%^[45]^. In PTC, pralsetinib had a 83% ORR (*n* = 6)^[45]^. Responses were also seen in RET fusion^+^ pancreatic cancer and intrahepatic bile duct carcinoma^[20]^. In the phase 1/2 trial of selpercatinib (NCT03157128) in patients with RET fusion^+^ solid tumors (LIBRETTO-001), a recommended phase 2 dose of 160 mg twice a day was established. Treatment-related toxicity was mostly in the low grade. Preliminary data from the selpercatinib trial showed an ORR of 68% in RET fusion^+^ NSCLC (*n* = 105). In treatment naïve RET fusion^+^ NSCLC, the ORR was 85%. The median duration of response was 20.3 months. The responses to pralsetinib and selpercatinib were observed in tumors with all RET fusion partners^[20,21,45]^. Thus, the results from pralsetinib and selpercatinib trials showed that these RET-specific TKIs gave high response rates comparable to those of ALK- and ROS1 fusion-targeted therapies in NSCLC at the tolerated doses and the responses were more durable than that of the multi-targeted TKIs with RET inhibitor activity.

## RET mutations resistant to multi-targeted and specific RET tyrosine kinase inhibitors

### RET mutations resistant to multi-targeted RET TKIs

A lesson learned from PTK-targeted therapies in chronic myeloid leukemia (BCR-ABL)^[46-48]^ and NSCLC (EGFR mutants, ALK and ROS1 fusions)^[49-53]^ is that the secondary mutations in the targeted PTK can cause resistance to the TKIs. By aligning MEN2/MTC-associated RET mutations with imatinib-resistant BCR-ABL residues, Carlomagno *et al.*^[54,55]^ found that the RET^V804L/M^ mutants at the gatekeeper residue and the RET^Y806C^ mutant at the hinge residue were resistant to vandetanib. Using the BaF3/KIF5B-RET cell model, we identified 14 RET kinase domain mutations resistant to cabozantinib, vandetanib, lenvatinib, and/or nintedanib^[27,56,57]^. These mutations were located in both drug-binding pockets and in the C-lobe outside of the drug-binding pocket. V804L/M mutations at the gatekeeper residue and Y806N mutation at the hinge region were pan-resistant to these drugs. Mutations at the C-lobe solvent-front G810 and N-lobe solvent front L730 residues had different profiles of resistance to these drugs depending on the substituting amino acid residues. Of particular interest was that the solvent-front RET^G810A^ variant, which was isolated as a vandetanib-resistant mutation, have gained slight sensitivity to the known type II inhibitor ponatinib and cabozantinib^[56,57]^. Two acquired vandetanib-resistant RET kinase domain mutation cases have been reported in NSCLC. The first was a CCDC6-RET fusion^+^ metastatic lung adenocarcinoma that developed adaptive resistance to vandetanib^[58]^. The resistant tumor had an acquired CCDC6-RET^S904F^ mutation. It was determined that the activating loop S904F mutation caused an allosteric effect that enhanced the basal kinase activity. The second reported case was a patient with CCDC6-RET fusion^+^ metastatic NSCLC^[59]^. The liver metastases responded to vandetanib initially, but progressed after 10 months. cfDNA analysis detected the presence of the CCDC6-RET^V804M^ gatekeeper mutation. In addition, a MTC patient with RET^M918T^ tumor was treated sequentially with sorafenib, vandetanib, cabozantinib, MGCD-516 and agerafenib. After disease progressed, a cfDNA assay revealed acquired RET^V804M^ gatekeeper mutation^[19]^.

### RET mutations resistant to RET-specific TKIs

As mentioned above, the potent, RET-specific TKIs pralsetinib and selpercatinib are effective against the RET^V804L/M^ gatekeeper mutants. An *in vitro* RET kinase assay showed that selpercatinib was ineffective against the RET^G810R^ solvent front mutant^[60]^. RET^G810^ residue is at the paralogous position of the C-lobe solvent front residue in EGFR^G796^, ALK^G1202^, ROS1^G2032^ and NTRK1^G595^/NTRK3^G623^. The EGFR^G796S/R^ mutants are resistant to the third-generation, EGFR^T790M^-sensitive EGFR inhibitor osimertinib^[61,62]^. The ALK^G1202R^ mutation was found in 21%-43% of acquired ALK mutations resistant to the second-generation, ALK^L1196M^ gatekeeper mutant-sensitive ALK TKIs certinib, alectinib and brigatinib^[63,64]^. ROS1^G2032R^ and the adjacent ROS1^D2033N^ mutations are resistant to crizotinib^[65]^. NTRK1^G595R^/NTRK3^G623R^ are resistant to entrectinib and larotrectinib^[66]^. Recently, we identified RET^G810C/S/R^ as selpercatinib-resistant mutations in a preclinical study^[67]^. Consistent with our preclinical finding, RET^G810C/S/R^ mutations were reported in a case of a KIF5B-RET fusion NSCLC and a RET^G810C^ mutation was reported in a case CCDC6-RET fusion NSCLC; both had adaptive resistant to selpercatinib^[22]^.

## Conclusions

Except for MTC and PTC, which have high rates of RET alterations, RET mutations and gene rearrangements have been observed at low frequencies in many types of human cancers. Gene rearrangements result in RET fusion oncogenes. Aside from a few well-characterized MTC-associated RET mutations, such as the RET^C634R/Y^ and RET^M918T^ variants, most of the recently identified RET mutations are of VUS. Moreover, the activating mechanisms of many MTC-associated RET mutations remain incompletely elucidated. For instance, it is believed that mutations of cysteine residues in the CRD lead to receptor dimerization via formation of intermolecular disulfide bonds^[4]^. However, experimental evidence to support this notion has yet to be reported.

The high ORR of RET-selective TKIs pralsetinib and selpercatinib in RET-altered cancers unequivocally demonstrates the RET dependence of tumors carrying these RET oncogenes. This observation has led to the proposal of molecular classification of RET-driven tumors as “retoma”^[68]^. In our view, RET fusion^+^ tumors could all be classified as retoma. Tumors with the RET^M918T^ mutant should be included as retoma because these tumors responded to pralsetinib and selpercatinib in clinical studies. Inclusion of tumors with other RET mutations in the absence of RET chromosomal rearrangement as retoma will await preclinical and clinical evaluations of these mutations.

It is anticipated that the potent RET-selective TKIs pralsetinib and selpercatinib will be approved by the United States FDA soon and thus become the first two approved RET-targeted therapy drugs. However, adaptive RET mutants resistant to selpercatinib have been found. Similar or novel RET mutants resistant to pralsetinib are predicted in addition to resistance caused by oncogene bypass mechanisms. Oncogene bypass mechanisms in solid tumors are multiple and may be influenced by tissue-specific tumor microenvironment. Intensive investigation is needed to overcome these targeted protein tyrosine kinase-bypass resistance mechanisms. For fusion protein tyrosine kinase-driven NSCLC that respond to potent, target-selective TKIs, on-target mutations appear to be a major adaptive resistance mechanism. While pralsetinib and selpercatinib are effective against the RET gatekeeper mutants, a lesson learned from protein tyrosine kinase-targeted therapies in NSCLC is that non-gatekeeper mutations will cause resistance to the new generations of TKIs. Pralsetinib and selpercatinib are no exception. Fortunately, TKIs of different chemical scaffolds can be developed to inhibit adaptive kinase mutants^[63,69]^. Therefore, the research and the pharmaceutical communities should continue to develop pipelines of RET-specific inhibitors. Pralsetinib and selpercatinib are an important milestone, but not the end of the journey, in developing RET-targeted therapy for retoma.

## Declarations

### Copyright

© The Author(s) 2020.
